# Short-term outcomes in robotic vs laparoscopic ileal pouch-anal anastomosis surgery: a propensity score match study

**DOI:** 10.1007/s00423-023-02898-1

**Published:** 2023-05-04

**Authors:** Sofoklis Panteleimonitis, Mahmood Al-Dhaheri, Mick Harper, Ibrahim Amer, Ayman Abdelhafiz Ahmed, Mohamed Abu Nada, Amjad Parvaiz

**Affiliations:** 1 University of Portsmouth, School of Health and Care Professions, St Andrews Court, St Michael’s road, Portsmouth, PO1 2PR UK; 2 Champalimaud Foundation, Av. Brasilia, 1400-038 Lisbon, Portugal; 3 Hamad Medical Corporation, Doha, Qatar

**Keywords:** Robotic colorectal surgery, Robotic pouch surgery, Robotic IPAA surgery

## Abstract

**Purpose:**

Laparoscopic ileal pouch-anal anastomosis (IPAA) surgery offers improved short-term outcomes over open surgery but can be technically challenging. Robotic surgery has been increasingly used for IPAA surgery, but there is limited evidence supporting its use. This study aims to compare the short-term outcomes of laparoscopic and robotic IPAA procedures.

**Methods:**

All consecutive patients receiving laparoscopic and robotic IPAA surgery at 3 centres, from 3 countries, between 2008 and 2019 were identified from prospectively collated databases. Robotic surgery patients were propensity score matched with laparoscopic patients for gender, previous abdominal surgery, ASA grade (I, II vs III, IV) and procedure performed (proctocolectomy vs completion proctectomy). Their short-term outcomes were examined.

**Results:**

A total of 89 patients were identified (73 laparoscopic, 16 robotic). The 16 patients that received robotic surgery were matched with 15 laparoscopic patients. Baseline characteristics were similar between the two groups. There were no statistically significant differences in any of the investigated short-term outcomes. Length of stay trend was higher for laparoscopic surgery (9 vs 7 days, *p* = 0.072)

**Conclusion:**

Robotic IPAA surgery is safe and feasible and offers similar short-term outcomes to laparoscopic surgery. Length of stay may be lower for robotic IPAA surgery, but further larger scale studies are required in order to demonstrate this.

## Introduction

Restorative proctocolectomy with ileal pouch-anal anastomosis (IPAA) is the main surgical treatment for patients with ulcerative colitis (UC) and familiar adenomatous polyposis (FAP), offering the benefit of avoiding a permanent stoma [1, 2]. However, there is a suggestion that in England the number of pouch procedures may be decreasing [3], possibly due to the associated problems of poor pouch function many patients experience.

Laparoscopic surgery has been increasingly utilised for pouch surgery [4] with a Cochrane review published in 2009 [5] and a randomised control trial in 2013 [6] supporting its feasibility and safety. Since then, there has been evidence demonstrating better cosmesis [6], improved fertility [7], lower blood loss [8], quicker recovery of bowel function [8] and faster recovery [9] with laparoscopic procedures when compared to open IPAA surgery. Nevertheless, the limitations of laparoscopic instruments make pelvic dissection and the proctectomy segment of the operation rather challenging. In England between 2012 and 2016, 60% of pouch surgery was still conducted through an open approach [3], and in the LapConPouch trial [6], a laparoscopic vs open pouch surgery trial, the conversion rate in the laparoscopic arm was 23.8%, possibly accounting for the reason why the number of pouch procedures in England has decreased. Alternative methods such as taTME have been described [10, 11], but the anatomy of performing the proctectomy from ‘back to front’ presents its own challenges. Robotic surgery addresses the limitations of laparoscopic instruments and is performed with the same anatomical approach as open and laparoscopic surgery. It is well established as an alternative method of operating for rectal cancer, with large multinational case series published in the literature [12, 13]. However, there are only a handful of small-scale studies for robotic vs laparoscopic IPAA surgery [14–18]. These studies are summarised in two recently published systematic reviews [19, 20], which conclude that more data is required.

This study aims to add to the current body of evidence comparing laparoscopic and robotic IPAA surgery and support its safety and feasibility.

## Materials and methods

A retrospective analysis of prospectively maintained databases was conducted for this study. Consecutive patients from 3 centres, for 3 different countries (England, Portugal and Qatar), who received elective minimally invasive proctocolectomies or completion proctectomies with IPAA reconstruction between 2008 and 2019 were identified and included in this study. The common denominator is that all cases were performed or supervised by a single surgeon with extensive experience in both laparoscopic and robotic colorectal surgery. The robotic cases were propensity score matched to laparoscopic cases to obtain comparable cohorts.

Patients were prepared for surgery according to each regional institutions policy. Patients with UC, FAP and confirmed colorectal adenocarcinoma (either on a background of UC or FAP) were included. The decision as to whether to perform the procedure laparoscopically or robotically depended on availability of the robotic platform, which was the preferred approach when available. Surgical dissection was similar for both laparoscopic and robotic procedures with a standardised fully robotic technique applied for all robotic surgeries as taught in the European Academy of Robotic Colorectal Surgery (EARCS) programme [12]. A fully robotic approach was applied in both the proctocolectomies and completion proctectomies. Dissection was completed by means of minimally invasive surgery, with an extraction site used for specimen delivery and pouch formation. The requirements for anonymisation of personal dataset by the Data Protection Act 1998 were satisfied. According to the Health Research Authority (HRA), this study did not require their approval due to its status as a clinical audit.

### Surgical procedure

#### Robot-assisted IPAA

##### Two stage proctocolectomy and ileoanal pouch

**Fig. 1:**
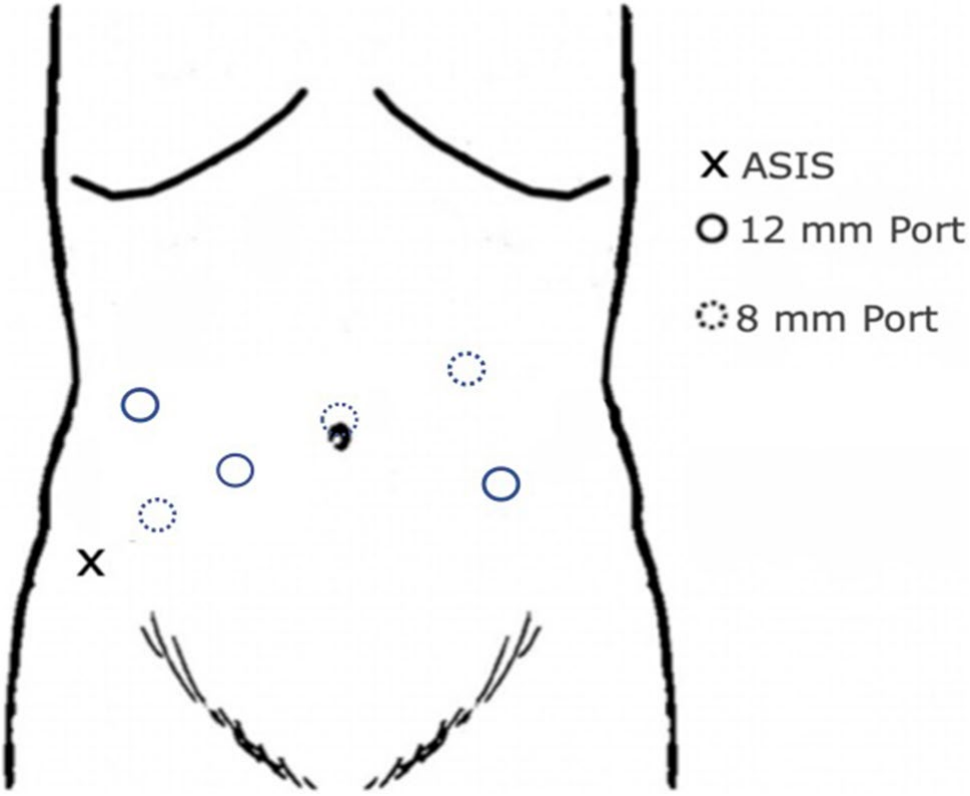
Port placement in robotic proctocolectomy and ileoanal pouch

All procedures were performed using 4th generation da Vinci Xi platform by Intuitive Surgical™. Three 8 mm and one 12 mm robotic ports were used for the surgery. Additional two assistant ports were also used to complete the procedure (Fig. 1). Extraction site for retrieval of specimen was made with 5 cm supra pubic incision. After creation of ileoanal stapled J pouch, protective loop ileostomy was created at the right iliac fossa.

Following insertion of ports and creation of pneumo-peritoneum, dissection starts at the sacral promontory with isolation, ligation and division of inferior mesenteric artery (IMA) and inferior mesenteric vein (IMV). Rectal mobilisation is performed in the TME plane protecting the hypogastric nerve plexus. The rectal tube is divided just above the anal canal at 4–5 cm from the anal verge using da Vinci 45 or 60 mm green load stapler.

Dissection continues with the mobilisation of the splenic flexure and left transverse colon. At this stage, robotic boom is rotated to perform right colonic mobilisation; the ileocolic artery and vein, right colonic vessels and middle colic artery branches are isolated, ligated and divided to complete the mobilisation of right and transverse colon. The specimen is delivered with 4–5 cm incision at the supra pubic area using Alexis wound protector.

Following the delivery of the whole specimen, a standard 18–20 cm stapled ileoanal J pouch is fashioned extracorporeally. Ileoanal anastomosis is performed using a 28-mm CEA circular stapler under the view. Flexible endoscopy is performed to check for leak test and integrity of the pouch. RIF protective ileostomy is performed at the previously marked right iliac fossa site.

The temporary ileostomy was closed if water soluble contrast enema performed at 2 months did not show anastomotic leakage or stenosis. Through incision in the right iliac fossa, side to side anastomosis was performed using a linear stapler [Covidien or Ethicon Inc., NJ, USA]. The wound was partially closed according to purse-string closure or simple closure according to the surgeon preferences.

#### Ileoanal pouch formation in patients with previous subtotal colectomy

Robotic port placement is standardised for the performance of proctectomy and ileoanal pouch formation in patients who had undergone previous emergency subtotal colectomy.

Following the creation of pneumo-peritoneum, the rectal stump is identified and mobilised in the TME planes down to the pelvic floor. Care is taken to identify and protect the hypogastric nerve plexus. The rectal tube is divided just above the sphincters using 45–60-mm green load da Vinci stapler. Specimen is extracted through the suprapubic 5 cm incision using Alexis wound protector.

Previously created ileostomy is mobilised, and standard 18–20 cm stapled J pouch is fashioned extracorporeally. Ileoanal pouch anastomosis is performed using a 28-mm circular stapler with protective loop ileostomy created at the same site of the right iliac fossa.

### Data collection and outcome assessment

The baseline characteristics and short-term surgical outcomes of patients receiving elective laparoscopic and robotic IPAA surgery were analysed. Baseline characteristics analysed included diagnosis, age, BMI, gender, American Society of Anaesthesiologist (ASA) grade, operation performed and previous abdominal surgery. Perioperative data included operative time, estimated blood loss (EBL) and conversion to open (defined as any incision needed to either mobilise the colon or rectum or ligate the vessels). Post-operative clinical data examined included length of stay (LOS), 30-day readmission, 30-day reoperation, Clavien-Dindo complication ≥ 3 and 30-day mortality.

### Statistical analysis

Once collated, cleaned and checked, the data was analysed using IBM SPSS version 26 (SPSS Inc., Chicago, IL, USA). The robotic cases were propensity score matched (PSM) to laparoscopic cases. The variables used to calculate the propensity score matching (PSM) were as follows: gender, previous abdominal surgery, ASA grade (I, II vs III, IV) and procedure performed (proctocolectomy vs completion proctectomy). PSM variable selection was based on what the authors believed to be of most clinical significance. Propensity scores were calculated via logistic regression analysis by applying the Propensity Score Matching function on SPSS version 26 with the match tolerance set to 0.

Non-parametric data was expressed as median with interquartile range and parametric data as mean with standard deviation. Baseline and clinical characteristics were compared using *χ*^2^ test or Fisher’s exact test for categorical variables, Mann-Whitney *U* test for non-parametric continuous variables and *t* test for parametric continuous variables. *P* values of < 0.05 were considered statistically significant.

Finally, univariate binary logistic regression analysis was performed on all patients receiving elective minimally invasive IPAA surgery to assess whether surgical approach (robotic or laparoscopic) affected morbidity (defined as the presence of any of the following outcomes: 30-day reoperation, Clavien-Dindo complication ≥ 3, 30-day readmission, LOS > 14 days). Following this, a multivariate model was applied where surgical approach was adjusted for all clinically relevant variables including age, gender, BMI, ASA grade (I–II vs III–IV), previous abdominal surgery and procedure performed. The constant was included in the analysis model and data is presented as odds ratio, 95% confidence interval and *p* value.

## Results

A total of 89 patients were identified (73 laparoscopic, 16 robotic) whose data was included in this study. The 16 robotic cases were propensity score matched with 15 laparoscopic cases to reduce the effect of confounding factors.

### Baseline characteristics

There were no differences in any of the baseline characteristics between the two groups as demonstrated in Table 1. PSM produced near perfect matches for gender, previous abdominal surgery, ASA grade and procedure performed. Proctocolectomies accounted for 5 (33.3%) and 5 (31.3%) of the laparoscopic and robotic IPAA procedures respectively, while UC was the primary diagnosis for the majority of cases in both groups (lap: 13 (86.7%), rob: 14 (87.5%)).

**Table 1:** Baseline characteristics of patients havening minimally invasive IPAA surgery

	Laparoscopic (*n* = 15)	Robotic (*n* = 16)	*p* value and test
Diagnosis			
UC	13 (86.7%)	14 (87.5%)	0.135 *χ*^2^
FAP	0	2 (12.5%)	
Cancer	2 (13.3%)	0	
Sex			
Male	7 (46.7%)	8 (50%)	0.853 *χ*^2^
Female	8 (53.3%)	8 (50%)	
ASA grade			
1 or 2	14 (93.3%)	14 (87.5%)	1.000 Fisher’s
3 or 4	1 (6.7%)	2 (12.5%)	exact
Previous abdominal surgery	10 (66.7%)	11 (68.8%)	0.901 *χ*^2^
Procedure			
Proctectomy	10 (66.7%)	11 (68.8%)	0.901 *χ*^2^
proctocolectomy	5 (33.3%)	5 (31.3%)	
Age	39.33 (± 14.96)	36.63 (± 11.42)	0.578 *t* test
BMI	24.81 (± 3.50)	25.84 (± 4.15)	0.460 *t* test

*UC*, ulcerative colitis; *FAP*, familiar adenomatous polyposis; *ASA*, American Society of Anaesthesiologist; *BMI*, body mass index

### Short-term outcomes

The short-term outcomes of the two groups are summarised in Table 2. There was only one conversion and this was in the laparoscopic group (*p* = 0.484). There was no 30-day mortality in either group. The short-term outcomes were largely similar and there was no statistically significant difference in any of the investigated short-term outcomes between the two cohorts. Length of stay trend was lower in the robotic group but did not reach statistical significance (7 vs 9 days, *p* = 0.072).

**Table 2:** Short-term outcomes of patients having minimally invasive IPAA surgery

	Laparoscopic (*n* = 15)	Robotic (*n* = 16)	*p* value and test
Conversion	1 (6.7%)	0	0.484 Fisher’s exact
30-day readmission	4 (26.7%)	3 (18.8%)	0.685 Fisher’s exact
30-day reoperation	1 (6.7%)	0	0.484 Fisher’s exact
Clavien-Dindo complications	IIIb: 1 (6.7%)	0	0.484 Fisher’s exact
30-day mortality	0	0	
Operation time (minutes)	248.67 (± 66.83)	241.25 (± 79.72)	0.780 *t* test
EBL (ml)	0 (0–50)	7.5 (0–12.5)	0.945 Mann-Whitney *U*
LOS (days)	9 (7–10)	7 (5–8.75)	0.072 Mann-Whitney *U*

*EBL*, estimated blood loss; *LOS*, length of stay

There was one reoperation in the laparoscopic group, which was a laparoscopic washout due to a collection. There were four readmissions in the laparoscopic group and three in the robotic group. All readmissions were due to either high stoma output or abdominal pain, with one patient having a wound infection. The only Clavien-Dindo complication ≥ 3 was the reoperation discussed.

### Logistic regression analysis

Univariate logistic regression analysis of all 89 cases showed that surgical approach did not affect morbidity for the participants in this study. This was still the case in multivariate analysis when other clinically relevant factors were adjusted for (age, gender, BMI, ASA grade (I–II vs III–IV), previous abdominal surgery and procedure performed). Table 3 summarises the described findings.

**Table 3:** Logistic regression for morbidity of all 89 patients having minimally invasive IPAA surgery

	Univariate				Multivariate			
	OR	95% CI lower	95% CI upper	*p* value	OR	95% CI lower	95% CI upper	*p* value
Robotic	0.725	0.211	2.491	0.609	0.737	0.203	2.674	0.642
Sex (male)	0.680	0.273	1.692	0.407	0.701	0.272	1.806	0.462
Age	1.010	0.972	1.049	0.617	1.012	0.970	1.056	0.587
BMI	0.934	0.827	1.055	0.275	0.932	0.819	1.060	0.282
Previous abdominal surgery	0.919	0.365	2.310	0.857	1.397	0.247	7.908	0.705
Procedure (proctectomy)	0.765	0.309	1.891	0.562	0.602	0.109	3.321	0.561
ASA (1–2 vs 3–4)	0.636	0.100	4.040	0.631	0.756	0.102	5.603	0.784

*ASA*, American Society of Anaesthesiologist; *BMI*, body mass index

## Discussion

In contrast to robotic surgery for colorectal cancer, there is very limited evidence investigating the clinical outcomes of robotic pouch surgery. In our study, we demonstrate that robotic IPAA surgery is safe and feasible with comparable short-term clinical outcomes to that of laparoscopic surgery. In addition, in the examined cohort, there was a trend towards a reduced length of stay in the robotic group, which is in agreement with other previous studies comparing robotic to laparoscopic IPAA surgery [17, 18].

The robotic platform offers a stable 3-D high-definition camera and wristed instruments with 7 degrees of freedom. The proctectomy part of pouch surgery can be technically challenging, and the adjuncts of robotic surgery are ideally suited for it. Its application in rectal cancer surgery is spreading in accelerated pace [12], and a handful of small volume studies have been published comparing robotic vs laparoscopic pouch surgery [14–18], summarised in a systematic review by Flynn et al. [19]. The number of robotic pouch procedures in these studies ranges between 16 and 74 cases. Our series is equally low in volume but offers the advantage of a comparison between equally matched cohorts as well as performing a logistic regression analysis of all available data to investigate the effect of robotic procedures on morbidity. As discussed by Flynn et al. [19], the main limitations of the available literature are the heterogeneity of the data and quality of the reported studies. Two of the five studies have only been published as conference abstracts [16, 18], offering only limited information on the study design. Some of the studies included cases where a completion proctectomy and end ileostomy was performed rather than an IPAA, and in other studies, there are significant differences between the two cohorts in terms of diagnosis (UC vs FAP) and procedures (proctocolectomy vs completion proctectomy) performed [19]. These differences potentially skew the outcomes of the reported studies. Moreover, in the reported literature, there is considerable variation on how the colectomy segment of the robotic proctocolectomies was performed, which could have been performed laparoscopically (hybrid approach), lap assisted or robotically (total robotic) [19]. In our study, all robotic cases were totally robotic, and no hybrid cases were done; this approach reduces cost since it avoids opening two sets of instruments and utilises the benefits of the robotic platform for the whole procedure.

In our study population, operation time was similar in the two groups. This is contrast to other studies that have shown larger operation times for the robotic cohort [14–18]. We speculate that this is because all procedures were performed or supervised by a surgeon with vast experience in robotic surgery that has fully overcome his learning curve [21]. Even though it was initially accepted that operation times were longer with robotic when compared to laparoscopic colorectal surgery, more recent studies have demonstrated similar operation times between robotic and laparoscopic rectal surgery [22–25], and some have even shown shorter operation times in the robotic cohorts [26–29]. This is potentially due to the improvements of the latest robotic systems (X and Xi), which limit arm clashing and reduce docking time [30]. Additionally, as surgeons overcome their learning curve in robotic surgery, they become faster and in difficult cases can even reduce their operative time when compared to laparoscopy by utilising the advantages the robotic platform has to offer [31]. Furthermore, the presented studies LOS is comparable to that of the literature (rob vs lap: 7 vs 9 days), with Rencuzogullari et al. [15] and Marino et al. [16] demonstrating similar LOS in both cohorts (rob vs lap: 7.8 vs 9 [15]; 8.7 vs 9.2 [16] days).

In our study, median LOS was shorter in the robotic cohort, although it was not statistically significant (7 vs 9 days, *p* = 0.072). Considering the small sample size, there is a realistic chance that there is a type 2 error and that robotic pouch surgery can indeed help reduce LOS. This is supported by Lightner et al. [17] and Elias et al. [18], with both studies reporting improved LOS in their robotic arms [17, 18]. The reason behind this might be that robotic surgery can enable more precise surgery in the pelvis, resulting in less tissue trauma, fewer complications and consequently a lower LOS.

Conversion rate, 30-day readmission, 30-day reoperation, Clavien-Dindo ≥ 3 complications and EBL were similar in the two cohorts. Furthermore, univariate and multivariate logistic regression showed that surgical approach (robotic or laparoscopic) was not found to affect morbidity. This is similar to the previous studies that also did not report any major differences in these outcomes between the two approaches [19].

The main strengths of our study lie on the standardisation of operative approach between all robotic and laparoscopic cases [12] and the fact that data was collected from multiple centres from different countries therefore increasing its external validity. Furthermore, due to the PSM, the two cohorts were evenly matched in terms of baseline characteristics, limiting the effect of cofounding factors.

Acknowledging its limitations, our study is a retrospective analysis, it is of small sample size and almost all of the laparoscopic procedures pre-dated the robotic ones. The fact that all laparoscopic studies pre-dated the robotic cases can act as a potential cofounder. However, it should be noted that this could confound the results either way. Although theoretically any improvements in the robotic cohort could be attributed to the increasing experience of the lead surgeon or improvements in the unit, the robotic cohort is much smaller and therefore it could be argued these are cases early on the learning curve. Another point worth mentioning is the fact that all cases were done or closely supervised by a very experienced in both laparoscopic and robotic surgery colorectal surgeon offers the advantage of standardisation of treatment but can reduce the external validity of the results. Furthermore, the small sample size of the study increases the chance of type 2 error, although, with the exception of operation time, the results are very similar. One way to reduce the risk of type 2 error would be to perform 1:2 or 1:3 propensity score matching. However, considering the number of laparoscopic cases to choose from is also relatively low (*n* = 73), this would compromise the matching process and result in less evenly balanced cohorts. The main drawback of this study is the lack of functional data (urological, sexual and pouch function), lack of patient reported outcomes, lack of fertility data and lack of long-term pouch data such as pouch failure rates or pouchitis. Additionally, estimated blood loss was not compared as it is generally poorly recorded and hence a poor indicator for assessing quality of surgery. It could be that the technical advantages the robotic system has to offer are more likely to lead in differences on those outcomes rather the short-term outcomes examined in this study. This is because the precision in dissection offered in robotic surgery could lead to better preservation of the pelvic nerves (leading to improved functional outcomes) and reduce trauma in the pelvis, thus reducing sub-clinical leaks or pelvic sepsis that, even though do not necessarily affect short-term outcomes, can have a detrimental effect on long-term pouch outcomes.

In summary, our study supports the feasibility and safety of robotic pouch surgery and contributes to the current body of evidence comparing robotic and laparoscopic IPAA surgery. Further larger scale studies are required comparing those two techniques in order to be able to produce more meaningful meta-analyses. Finally, it is important to collect functional, fertility and long-term pouch function data since these outcomes are important to patients and the robotic platform is more likely to make a difference in these outcomes.
